# Clinical Outcome of Laparoscopic Intersphincteric Resection Combined with Transanal Rectal Dissection for T3 Low Rectal Cancer in Patients with a Narrow Pelvis

**DOI:** 10.1155/2011/901574

**Published:** 2011-12-29

**Authors:** Kimihiko Funahashi, Hiroyuki Shiokawa, Tatsuo Teramoto, Junichi Koike, Hironori Kaneko

**Affiliations:** ^1^Department of Gastroenterological Surgery, Toho University Medical Center, Omori Hospital, 6-11-1 Omorinishi Otaku, Tokyo 1438541, Japan; ^2^Department of Surgery, JyuJyo Hospital, Chiba prefecture, Japan

## Abstract

*Purpose*. The purpose of this study was to analyze the safety and feasibility of laparoscopic intersphincteric resection (ISR) combined with transanal rectal dissection (TARD) for T3 low rectal cancer in a narrow pelvis. *Methods*. We studied 20 patients with a narrow pelvis of median body mass index 25.3 (16.9–31.2). Median observation period was 23.6 months (range 12.2–56.7). *Results*. Partial, subtotal, and total ISR was performed in 15, 1, and 4 patients, respectively. Median duration of TARD was 83 min (range 43–135). There were no major complications perioperatively or postoperatively. Surgical margins were histologically free of tumor cells in all patients, and there was no local recurrence. Excluding urgency, frequency of bowel movements, and incontinence status improved gradually after stoma closure. *Conclusion*. Laparoscopic ISR combined with TARD is technically feasible for selective T3 low rectal cancer in patients with a narrow pelvis.

## 1. Introduction

Intersphincteric resection (ISR) to preserve anal sphincter function for low rectal cancer extending into the anal canal was reported by Schiessel et al. in 1994 [1]. The feasibility of ISR has been demonstrated by surgeons since that time; it is now technically possible to use ISR to remove low rectal cancer with preservation of anal sphincter function with a satisfactory oncologic outcome [2, 3]. Recently, the clinical outcome of ISR as a laparoscopic approach (laparoscopic ISR) has been reported, but laparoscopic ISR for patients with bulky low rectal cancer remains challenging. Particularly for T3 tumors in patients with a narrow pelvis, it is important to achieve a low local recurrence. Total mesorectal excision (TME), negative circumferential margin (CFM), and tumor free surgical margin are prerequisites regardless of approach of ISR. Conversion to open operation in laparoscopic ISR may influence prognosis, as is the case in laparoscopic surgery for rectal cancer [4]. We have shown that transanal rectal dissection (TARD) performed prior to the abdominal phase of the operation is very useful for an adequate oncologic resection in laparoscopic ISR for T3 low rectal cancer in patients with a narrow pelvis [5]. The purpose of this report is to evaluate the safety and feasibility of TARD to achieve laparoscopic ISR for T3 low rectal cancers in patients with a narrow pelvis.

## 2. Patients

Preoperative staging evaluation included digital rectal examination, barium enema, colonofiberscope with biopsy, computed tomography (CT), magnetic resonance imaging (MRI), and transanal ultrasound (TAUS). The patients were excluded when preoperative examination showed the following findings: multiple metastases in distant organs, direct invasion into adjacent organs (clinical T4), involvement of lateral lymph nodes, and invasion into the external anal sphincter or/and levator ani. We studied 20 patients (5 women, 15 men) with a median age of 66 years (range 42–77 years) between April 2006 and December 2009. In all patients the tumors were bulky in nature, and narrow pelvic dimensions were expected for laparoscopically assisted pelvic floor dissection on the basis of radiographic findings of barium enema, CT, and MRI. Preoperative CRT was performed in 2 men out of the 20 patients. Finally, preoperative TNM staging of the 20 patients was T3 N0 M0 in 8, T3 N1 M0 in 9, T3 N2 M0 in 2, and T3 N3 M1 in one. Median body mass index was 25.3 kg/m^2^ (range 16.9–31.2 kg/m^2^) (Table 1). The patients were observed for a median of 23.6 months (range 12.2–56.7 months).

## 3. Surgical Technique

**Figure 1:**
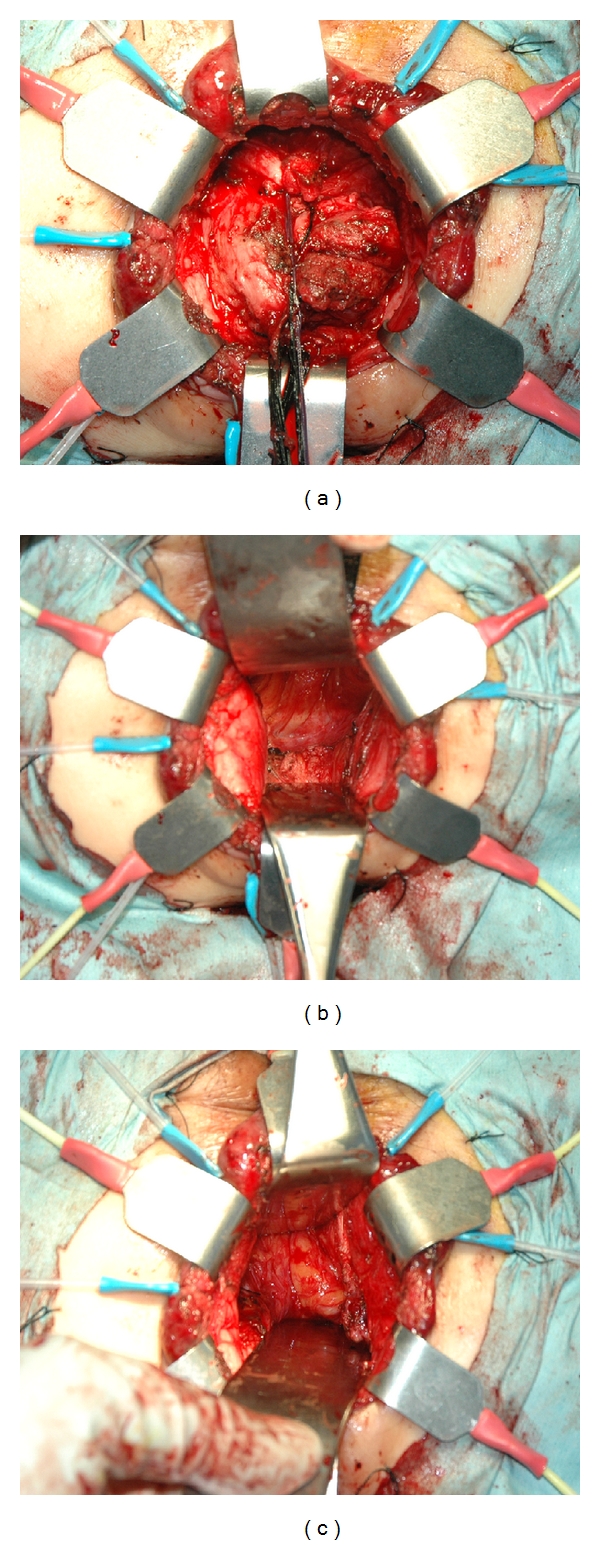
Transanal rectal dissection for a male patient with T3 low rectal cancer. A circular incision of the rectum was performed by closing the cut end of the rectum (a). The rectum including the tumor was mobilized proximally by exposing the levator ani (b, c).

Surgical technique regarding TARD has been described previously [5]. The operation is performed in the Lloyd-Davies position. Prior to the laparoscopically assisted abdominal phase, the anal portion of the operation is initiated. First, TAUS is performed to confirm the depth of invasion. If TAUS shows tumor invasion to the external sphincter and/or the levator ani, an abdominoperineal resection (APR) should be chosen as the surgical procedure. The anal canal is exposed with a self-holding retractor (Lone Star Retractor, Lone Star Medical Products Inc., Houston, TX, USA). The distal side at the lower margin of the tumor is then closed with purse-string sutures under direct visualization, followed by irrigation of the anal canal with 5% povidone-iodine. This step is important for preventing cancer cell dissemination in the surgical field. The division of the rectum is then initiated posteriorly at least 2 cm distal to the tumor margin. A circular incision of the rectum is performed by closing the cut end of the rectum with an interrupted suture, and mobilization of the rectum, including the tumor, is continued proximally by exposing the levator ani. Invasion of tumor cells on the dissected plane (the external sphincter or/and the levator ani) should be evaluated by microscopic examination of a frozen-section specimen histologically whenever mobilization of the rectum is not easy. If any findings of tumor invasion into the dissected plane are found, the procedure should be immediately converted to abdominoperineal resection (APR). Division and mobilization of the rectum, including the mesorectum, is performed until the peritoneal reflection on the anterior side, and up until the sacral promontory beyond the rectosacral ligament, is nearly reached posteriorly. Finally, a Lap disc mini (HAKKO Group, Japan) is adapted to the anal canal to maintain pressure during laparoscopy (Figure 1).

**Figure 2:**
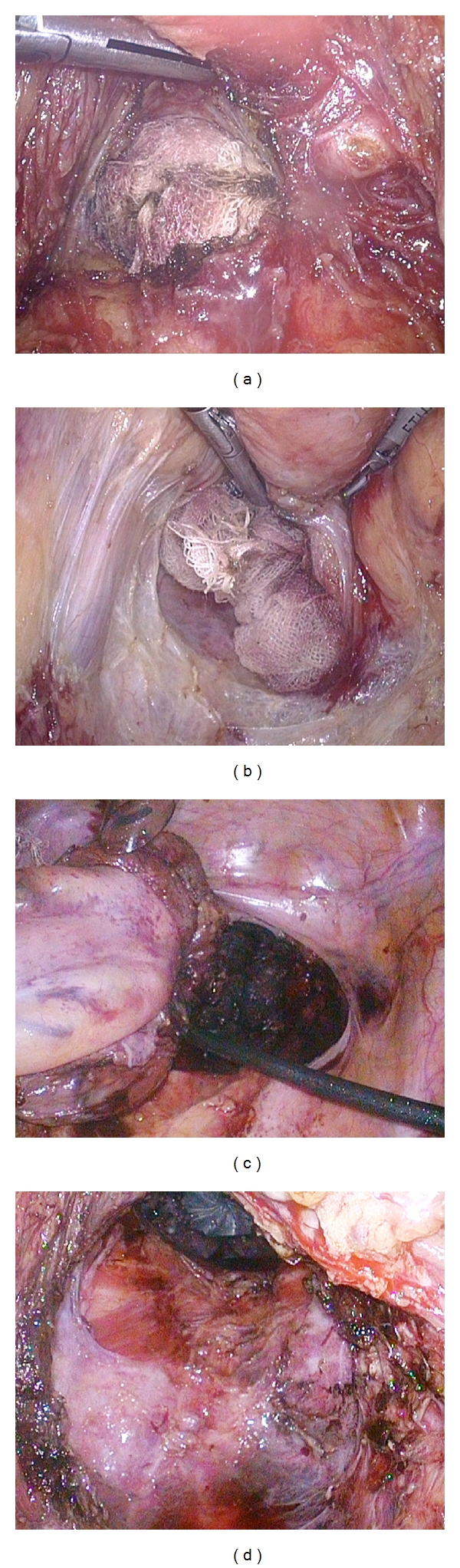
Laparoscopic procedure combined with transanal rectal dissection. The gauze that was placed on the dissected plane as a landmark was able to be identified through the peritoneum on the anterior side on the rectum. It was relatively easy to dissect Denonvillier's fascia and expose the seminal vesicles and prostate gland (a). On the posterior side of the rectum, it was possible to mobilize the lower rectum and mesorectum from the sacrum on the separated plane between the visceral and parietal endopelvic fascia through the anus (b). The lateral ligaments of the rectum were gradually divided with a harmonic scalpel from the inner limit of the inferior hypogastric nerve fibers. The rectum, including the total mesorectum, was completely removed from the pelvic floor (c, d).

Regarding the laparoscopic procedure, a camera port is inserted in the paraumbilical zone with a trocar, and an operative port in the mid-lower abdominal region, and two additional operative ports in the left and right Mc Burney's point are inserted. On routine intra-abdominal exploration, the gauze that is placed on the dissected plane as a landmark can be identified through the peritoneum on the anterior side of the rectum. The sigmoid and descending colon are mobilized completely from the subretroperitoneal fascia to ensure that the subsequent coloanal anastomosis is free of tension. The sigmoid colon and its mesentery are then removed, the lymph nodes around the inferior mesenteric artery are dissected with a harmonic scalpel, and the inferior mesenteric artery is ligated at a high level with an endoclip. It is relatively easy to dissect Denonvillier's fascia and expose the seminal vesicles and prostate gland or the posterior wall of the vagina on the anterior side and to mobilize the lower rectum and mesorectum from the sacrum on the separated plane between the visceral and parietal endopelvic fascia through the anus. The lateral ligaments of the rectum are gradually divided with a harmonic scalpel from the inner limit of the inferior hypogastric nerve fibers, and the rectum, including the total mesorectum, is completely removed from the pelvic floor. The colon and rectum are pulled out of the umbilical wound and are resected. A coloanal anastomosis is transanally performed by hand suturing. Finally, a diverting ileostomy is created. The diverting ileostomy is reversed three to six months after surgery (Figure 2).

### 3.1. Functional Assessment

Sphincter function was evaluated clinically in 3, 6, and 12 months after stoma closure. The patients were questioned about frequency of bowel movements, ability to defer defecation for 15 minutes (urgency), and satisfaction of defecation status using visual analogue scale (VAS). Continence status was determined according to the classification of Wexner incontinence score (WIS).

## 4. Results

The numbers of patients undergoing partial, subtotal, and total ISR were 15, 1, and 4, respectively. There was no conversion to an open operation. The median duration of TARD procedure was 83 min (range 43–135 min) and was longer in males than in females (81 min versus 89 min). Although there were no major complications perioperatively or postoperatively, anastomotic stenosis in two male patients, bowel obstruction in one male patient, and pelvic abscess formation in one female patient occurred postoperatively. Morphologically, the median maximum tumor size was 42 mm (15–75 mm), and the median circumferential rate of tumor was 66% (27.7–90.0%). The average distance from the rectal stump was 16 mm (range 7–40 mm), and circumferential and distal margins were histologically free of tumor cells in all patients. Pathological response grading following preoperative CRT performed for two patients was grade 2 and grade 1b, respectively. Finally, postoperative pathological staging was ypT2N0M0 in one, ypT3N0M0 in one, pT2N0M0 in 4, pT2N1M0 in 2, pT3N0M0 in 7, pT3N1M0 in 2, pT3N2M0 in 2, and pT3N2M1 in one patient. The median number of evaluated lymph nodes was 12.5 nodes. Distant organ metastasis developed in 2 patients, but there was no local recurrence.

 Eighteen out of 20 patients received stoma closure excluding one with distant metastasis and one who did not want stoma closure. In this study sphincter function was investigated for twelve out of 18 patients in 3, 6, and 12 months after stoma closure. Half ten patients, experienced nine and more bowel movements a day, 8 (80%) complained urgency, and 8 (80%) reported five or less VAS in three months after stoma closure. In twelve months after stoma closure, the rate of the patients who experienced nine and more bowel movements a day and reported five or less VAS decreased to 20% and 17%, respectively, but nine (75%) complained urgency. Regarding continence status, the rate of the patients answered ten and more WIS in three months and twelve months after stoma closure were 50% and 33%, respectively (Table 2).

## 5. Discussion

ISR has been shown to preserve anal sphincter function and provide an adequate oncologic resection for low rectal cancers since Schiessel's first report in 1994. The pooled rate of local recurrence was 0–31%, with an average 5-year survival of 81.5%, in an evaluation of the experience of 13 centers and 612 patients by Tilney and Tekkis [2]. Recently, clinical outcomes of ISR as a laparoscopic approach have been reported, but laparoscopic ISR for bulky low rectal cancer is challenging, especially for T3 low rectal cancer in patients with a narrow pelvis. Laurent et al. [6] made a comparison between 110 patients undergoing the laparoscopic approach and 65 patients undergoing an open approach and reported a satisfactory outcome of laparoscopic ISR, with a 5-year disease-free survival of 70% and a 5-year local recurrence of 5%. Fujimoto et al. [7] also noted the advantages of laparoscopic ISR in their evaluation of 35 patients with low rectal cancer. However, in these reports the influence of narrow pelvic dimensions on outcomes of laparoscopic ISR was not described. Also, Akasu et al. [8] reported that local control for T3 tumors was difficult as compared with T1-T2 tumors. In our study, only patients with T3 low rectal cancer and a narrow pelvis were included in the analysis. With consideration of a good oncologic outcome with a low recurrence rate after surgery for T3 low rectal cancer, some prerequisites are necessary regardless of the ISR approach: TME, negative CFM, and tumor-free surgical margins. In most prior studies, pathological TNM stage and T stage were reported as important risk factors for prognosis. In addition, Akasu et al. [9] reported that the resection margin, focal differentiation, and serum CA 19-9 level were important risk factors of local recurrence in an evaluation of 120 patients with very low rectal cancer including 46 patients with stage III disease. In this study, preoperative radiographic examination demonstrated bulky tumor occupying the pelvis in all patients. Although preoperative CRT in order to decrease the volume of tumor and prevent local recurrence was performed only for two patients secondary to preference, the resection margin including the radial margin was histologically free of tumor in all patients including patients without preoperative CRT. Conversion to open operation impacted significantly on overall survival except when considering long-term disease-free survival with laparoscopic surgery for colorectal cancer [4]. This subject deserves more than a passing notice, and conversion to open operation should be avoided to prevent local recurrence in laparoscopic ISR as well. In general, the following risk factors for conversion to open operation from traditional laparoscopic surgery for rectal cancer were reported: obesity, bulky tumor, and bony pelvis. In laparoscopic ISR, these factors may make laparoscopically assisted pelvic dissection even more challenging because these factors further confine the surgical field, hindering visualization and retraction in a deep and narrow pelvis. Tekkis et al. [10] and Scheidbach et al. [11] reported a direct correlation between increasing body mass index and higher conversion rates for laparoscopic colorectal surgery. Bège et al. [12] and Yamamoto et al. [13] confirmed the correlation between body mass index and conversion rate. In this study, laparoscopic ISR without conversion to open operation was achieved for all patients, with a median body mass index of 25.3 kg/m^2^ and a median tumor circumferential rate of 66%.

In general, transanal manipulation for dissection of the tumor from the levator ani and external sphincter is performed after the abdominal phase of ISR, including the procedure described by Schiesel. On the other hand, Teramoto et al. [14] and Watanabe et al. [15] introduced per anum intersphincteric rectal dissection with direct coloanal anastomosis (PIDCA), a surgical technique for low rectal cancer performed before the abdominal phase. However, long-term outcomes with local recurrence at 31% were not satisfactory, the reasons for which are unclear [16]. Although Uchikoshi et al. [17] reported good clinical results with laparoscopic ISR combined with transanal manipulation prior to the abdominal phase for two patients with T2 very low rectal cancer and total colectomy for two patients with ulcerative colitis complicated by T1 colorectal cancer; feasibility for T3 low rectal cancer could not be evaluated due to the small number of patients. We also consider that TARD as the transanal procedure performed prior to the laparoscopically assisted abdominal phase is very useful to achieve a good oncologic result with a low local recurrence, when performed with laparoscopic ISR for bulky low rectal cancer, especially T3 low rectal cancer in patients with a narrow pelvis. In fact, we experienced neither major complication nor conversion to open operation in this study. For T3 tumors, a high local recurrence rate in patients without radiotherapy was reported by Tekkis et al. [10], but there was no local recurrence in selective patients with a narrow pelvis. However, this study was retrospective and limited by a short postoperative observation period (median 23.6 months). Exclusion of patients with T4 tumors with TAUS preoperatively may decrease local recurrence. In addition, TARD was able to dissect with adequate radial margins around the tumor under direct vision even if the tumor invaded near the levator ani and was considered to be effective for a good oncologic outcome. In this study, preoperative CRT decreased the volume of the primary tumor in one patient allowing for laparoscopic ISR. However, the other patient had Grade 1b cancer, and preoperative CRT was not considered to be effective for laparoscopically assisted pelvic floor dissection. While some researchers have reported a good correlation between the volume reduction rate of primary tumor and pathologic tumor response of preoperative CRT [18, 19], complete pathological response rate was reported to be only from 7% to 34.7% [20–22]. For some of the nonresponders, a histological reaction (fibrosis and/or edema) may have occurred in the rectum itself, and adjacent organs may have made pelvic dissection around the tumor more difficult.

Sphincter function after ISR impacts on quality of life of patients significantly. In this study, sphincter function was investigated for limited patients in 3, 6, and 12 months after stoma closure. Frequency of bowel movements and WIS improved gradually, but the fact that 75% of the patients complained urgency in 12 months after stoma closure can hardly be ignored. Although preoperative radiation therapy, volume of resected internal sphincter muscle, or gender was reported as poor risk factors of sphincteric dysfunction, these results could not be explained by these factors in this study [23–26].

In conclusion laparoscopic ISR will be widely adopted as an acceptable procedure to preserve anal sphincter function for low rectal cancer extending to the anal canal. Laparoscopic ISR combined with TARD is technically possible for selective T3 low bulky rectal cancer, and a satisfactory clinical outcome was achieved in this series.

## Figures and Tables

**Table 1:** Characteristics of patients.

Parameter	*N* = 20
Median age	66 (42–77)
Gender: male/female	15/5
Median body mass index (kg/m^2^)	25.3 (16.9–31.2)
Preoperative TNM staging	
T3N0M0	8
T3N1M0	9
T3N2M0	2
T3N3M1	1
ISR	
Partial/subtotal/total	15/1/4
Median duration of TARD (min)	83 (43–135)
Male	89 (50–135)
Female	81 (43–97)
Postoperative TNM staging	
ypT2N0M0	1
ypT3N0M0	1
pT2N0M0	4
pT2N1M0	2
pT3N0M0	7
pT3N1M0	2
pT3N2M0	2
pT3N2M1	1
Median tumor size (mm)	42 (15–75)
Median circumferential rate of tumor (%)	66 (27.7–90)
Median distal margin (mm)	22.5 (7–40)

ISR: intersphincteric resection.

**Table 2:** Sphincter function after stoma closure.

		3 months (*n* = 10) No. of patients (%)	6 months (*n* = 10) No. of patients (%)	12 months (*n* = 12) No. of patients (%)
Urgency		8 (80)	8 (80)	9 (75)

Frequency of bowel movements	≤3	1 (10)	3 (30)	3 (30)
	4-5	3 (30)	3 (30)	6 (60)
	6–8	0	1 (10)	1 (10)
	≥9	5 (50)	3 (30)	2 (20)

VAS	<5	8 (80)	4 (40)	2 (17)
	5–7	1 (10)	3 (30)	3 (25)
	≥7	1 (10)	3 (30)	7 (58)

WIS	<10	5 (50)	6 (60)	8 (67)
	≥10	5 (50)	4 (40)	4 (33)

VAS: visual analogue scale, WIS: Wexners' incontinence score.
